# Where Shall We Spend the Summer?

**Published:** 1875-08

**Authors:** 


### WHERE SHALL WE SPEND THE SUMMER?

In our last issue we considered this question, from the stand-point of an excursion to the mountains of New Hampshire and Vermont, lasting several days, and embracing numerous points of interest. These notes of travel we now continue.

No place interested us more than St. Johnsbury, Vermont, and if ample facilities existed for the accommodation of guests, we do not see why this charming locality should not be deemed a most desirable summer resort. The country is rolling and abundantly wooded, and the views from the surrounding hill-tops are rarely surpassed. Great hotels do not abound, but there is one very good one, the St. Johnsbury House, and there are accommodations for a moderate number of summer boarders. With the refinement and wealth and social surroundings which prevail at St. Johnsbury, added to the natural beauty of the place and the lavish adornment of art, it would seem only necessary to provide accommodations for summer visitors in order to secure them in troops. It was told us at Bethlehem that all the people of that little mountain town were getting rich by keeping summer boarders. If accommodations existed for 3,000 additional people at St. Johnsbury, we believe that number would flock thither on the simple announcement of the fact. This small army of temporary sojourners would leave very nearly half a million dollars in the place during the summer months, and would carry away millions in the form of renewed life and added vitality.

We were much interested in the great work done and in progress, by the family of Fairbanks, whose immense Scale works are situated here. We were courteously shown over the factories, and were allowed to witness the various manipulations. The method and system employed are wonderful, and the apparent intelligence of much of the machinery struck us as well-nigh marvellous. To the public-spirited members of the family of Fairbanks, St. Johnsbury clearly owes many of its most attractive features. To them the elegant Public Library—with its Art Gallery—is due. Here, among a number of art gems, may be seen Bierstadt's wonderful creation, "The Domes of the Yosemite," bought by the Fairbanks' of the LeGrande Lockwood estate, and presented to the town. As we left the place we remarked, "well, whatever else this town may lack, she certainly does not suffer for want of sterling, public-spirited men."

We take a long stride now, and find ourselves, June 10th, at Saratoga. Here we found the enormous hotels, the Grand Union,—owned by Mr. A. T. Stewart, of New York, and kept by Mr. J. H. Breslin; the United States, owned by Mr. Marvin, of Philadelphia, and under the management of Messrs. Tompkins & Perry; the Clarendon, by Mr. Chas. E. Leland, who is also the proprietor of the Delevan, Albany; Congress Hall, by Mr. Hathorne—all sounding the note of preparation for an anticipated active campaign. All said that the season was late, but they looked for a large business in July and August. Here, too, we were impelled to call at an establishment which has become widely celebrated, but which we had never visited—the "Remedial Institute" of the Drs. Strong. The house is delightfully situated, on an eminence a couple of blocks from Broadway, the fashionable street. The immediate surroundings of the "Institute" are rural and attractive. Piazzas, broad and ample, surround the house, and green trees lend their grateful shade. The lawn is spacious, and croquet-playing is well-nigh endless.

The "Institute" comfortably accommodates one hundred and fifty guests. It is heated by steam, and a perfect system of ventilation exists. The steam is always up and is turned on, in any part of the house, in the cold, wet, summer days, as well as in winter. Those great extremes of temperature which are the real bane of this latitude, are here intelligently guarded against. As a summer resort, the establishment has a wide popularity among a large class of intelligent men and women. The fashionable butterflies, of course, prefer the great hotels, with their display and excitement, their dissipation and their riotous living. Those who have aims and objects in life, who seek to live like responsible human beings, enjoy the quiet found at Dr. Strongs. Those who are feeble, find here all of good which Saratoga possesses—the springs, the walks, the drives, the pure air—and, besides, the many added appliances for the restoration of health, which education and experience can supply. More than this, the cultivated visitor finds here the very society which he would select from all the world :—people of brains, of taste, of accomplishments; people who read and reflect; people who have travelled and know the world of nature, of literature, of art and of science. Nine out of ten of those whom you will meet in a picture-gallery, are lovers of pictures; so, nine out of ten of those who become guests of Dr. Strong's house, are animated by the very thought which makes them pleasant companions and valuable acquaintances. This secures a homogeneous family, and a delightful, home-like atmosphere, especially attractive to the worn and tired stranger. For ourselves, we felt,—after going pretty thoroughly over the Remedial Institute and examining the baths, and all the numerous appliances for the treatment of disease,—that here indeed was the TRUE HOME of Saratoga, the real harbor of rest and comfort and relief from physical pain. Of the special methods employed here for the relief of human suffering, we hope to speak hereafter; we will now merely add that the Drs. Strong, father and son, are educated medical men, and that the great work in which they are engaged is conducted with intelligence, integrity and skill. They issue a very interesting pamphlet, which they mail free to applicants. From Saratoga, we proceeded to Richfield Springs, in Otsego County, long famous as a summer watering-place and health resort. We passed the night at one of the best hotels seen in all our travels—Baggs' Hotel, Utica. It is owned and managed by Messrs. Proctor and Chamberlain, and its table can hardly be surpassed for the perfection of its cooking. The "Spring House," at Richfield, has been purchased recently, by Mr. Thomas R. Proctor, of Baggs' Hotel. He had put the house and grounds in complete order, and has given the place a very tasteful appearance. There are accommodations for six hundred guests. The attractions of the vicinity are many and varied. The scenery is beautiful, and feeds the eye with all the variety of a charming landscape. The region is mountainous, and the village stands more than a third of a mile nearer the sky than the city of New York. From an observatory, seventy-five feet high, in the outskirts of the town, a marvellous panorama of lake and forest, and garden and farm, and carpet of green, is seen on every hand. We counted six sheets of water within a range of a mile or two. These lakes abound in trout and pickerel, and are well supplied with trim little boats for those who would skim the blue waters, or tempt the finny tribe from home and friends. It is a very garden-spot in its natural advantages, and has had the benefit of admirable cultivation by intelligent farmers. All its surroundings are healthful and attractive. To the invalid, the chief attraction must ever be its wonderful mineral springs. These are numerous; but the most famous of them all is the sulphur spring, on the grounds of the Spring House. The water is clean and cool, and gives off a very perceptible odor of sulphuretted hydrogen. The presence of sulphur is also evident to the taste; so evident indeed, as to be a little unpleasant at first. Yet the constant imbibers of the water declare that it becomes decidedly palatable after continued use. It is a gentle laxative, as may be inferred from the fact that its leading ingredient is sulphate of magnesia.

The discharge from the spring is increasing, and amounts to a stream three-fourths of an inch in diameter. All the world comes and partakes freely of this healing water. The excess is conducted to a large reservoir from which a supply is taken to be heated by steam for the bath-houses. These baths are wonderfully potent in many diseases. All blood-taints find here their sovereign remedy. In rheumatic affections the fame of this spring is probably unexcelled. Judge Charles Mason, of the Court of Appeals, declares that there is no such water in all the world for the cure of rheumatism, and that he has often helped people in on beds, who have gone out cured. In fact, the testimony to the value of this spring as a healing power, is enormous and irrefutable.

We conversed with Dr. N. Getman and Dr. W. B. Crain, resident physicians, and found them fully alive to the value of the water, when intelligently employed. Both of these gentlemen use it constantly in their practice, and recite cases in which the benefit resulting from its use was marked.

A very entertaining book, entitled "Richfield Springs and vicinity," was written by Dr. W. T. Bailey, and published last year by A. S. Barnes & Co. It gives much valuable information concerning Richfield, its surroundings, its attractions, its people and its mineral waters.

The sanitary arrangements and surroundings of the Spring House, under its new proprietor, Mr. Proctor, are unsurpassed by those of any hotel in America. With its potent Sulphur-waters, intelligently employed, there is no reason why this should not become the leading resort for invalids, as it has long been for pleasure-seekers. It may be said with emphasis, that Mr. Proctor knows "how to keep a hotel." He is a gentleman, and therein he possesses the first essential of success. He has travelled widely, and knows the wants and needs of travellers. He has had ample experience in the business to wear off all the crudities of inexperience, and to attune his quick intellect to the demands of people of high and low degree. He will make of the Spring House a grand success, for he will deal liberally and justly with all his guests.

There are several other hotels here, and many boarding-houses. Home comforts abound, and prices vary according to accommodations and locality. A tour of the village will, it is said, discover all grades, from $1 a day up. So far, we have seen nothing, in all our wanderings, which pleases us more than Richfield.

Our next call was at Binghampton. Here the chief attraction is Col. Dwight's elegant hotel, and the system of lovely cottages surrounding it. Whatever money can do to adorn and beautify a region upon which nature has been by no means niggardly, has been done. Parks and fountains and well-kept lawns are seen in all directions. Within, all is good taste and quiet elegance. The furniture of the hotel is superb and the conveniences are not surpassed in the grandest hotel in this metropolis. Col. Dwight is doing a noble work for his native town, as well as for tourists and travellers, and we are glad to be able to bear our testimony to the ability and good taste which he has exercised.

From Binghampton we pushed on to Niagara. The grand old cataract was unchanged. Twenty feet in depth of water was still breaking over the rock, with its unceasing thunder. The many-tinted rainbow still circled around the spray at its base. Nothing strikes the heart of man like Niagara.

We took our favorite walk through Prospect Park, and stood out over the mighty cataract, on the parapet of solid masonry. We could dip our cane in the very torrent itself as it broke over the rock and was dashed into mist below. Then we rode down the incline on the railway belonging to the Park, and stood on a level with the river beneath. Here, in the still waters, so lately torn into fragments by the terrible descent, was the boat to ferry us over to the Canadian side. The entire passage is made in front of the cataract, and from no other point of view, to our mind, is the sight as grand. Prospect Park is a well conducted institution. We have visited it many times, and have found it a delightful retreat, and a safe one, from the importunities of the multitude who live by what they can manage to extort, in one way and another—by devices far from creditable, sometimes—from visitors to Niagara. It is easy to avoid extortions, if people will but take with them to Niagara such common sense as nature has blessed them with. If they go to squander money, the task will not be found a difficult one. They have only to allow the first hackman to allure them into his carriage, and the work is done. He will carry them endless miles and show them the Dominion of Canada and the kingdoms of the earth; and the pocket must be a plethoric one which can withstand the havoc. Of all the follies perpetuated at the Falls, the carriage folly is the premium one. A person having the free use of his or her limbs, no more needs the aid of a horse to see the river from any desirable point, than he needs a balloon for the purpose. The task is a simple one and a cheap one. All that is really essential is the Prospect Park and its belongings, and Goat Island. By way of the first he is landed in Canada by boat, and a short walk on that side gives him the whole majestic view. Goat Island can be visited at leisure. You do not see actually the Falls from it, but you do see the rapids, the rush of mighty waters, in the presence of which you will tremble. The cost of these all-important visits is but trifling; a dollar or two at the most. For four shillings more you will be given tickets of admission for the whole summer.

Great reforms are going on at this famous resort. The Park Company exert a good influence constantly. The great hotel, the "International," has gone into the hands of tried and trusty men, who will frown upon all abuses. They are there as hosts, to protect their guests from fraud. They know the wants of the outside world, and are determined that those wants shall be fully met. The new proprietors are Mr. Gale, late of the Arlington, Washington, and Fuller, for many years connected with the Brevoort House, New York. They are experienced gentlemen, and we shall be surprised if they do not make the International the most popular hotel at Niagara. This much we are able to avouch, that those who seek their advice as to the best and least expensive means of "doing" the Falls need feel no anxiety. Whatever local hackman, or small dealer in Indian curiosities may suffer, the visitor and the stranger will be protected.

There is, it seemed to us, abundant room for a new hotel at Niagara, and there is a vacant spot upon which it should be placed. That spot is the little grove near the entrance of the Park. If that lot could be bought of the Park Company, and a hotel, costing, say, $75,000, could be built this year, it would be paid for in profits, during the Centennial season. If we were a hotel man we should go to Niagara to talk this matter over with Mr. D. T. Townsend, the enterprising President of the Prospect Park Company. Such a house, in such a spot, would, we think, prove to be the best Hotel property in America.

Long Branch welcomed us for a day. The proximity of this favored spot to New York makes all things lively during "the season." The great hotels, fronting the grand old ocean, were alive with well-dressed people. The Ocean House, kept by "Uncle Charles" Leland and Warren Leland, Jr., was apparently the central point, the most popular and populous of all the hotels. This fact is due to the fact that the Leland family are born hotel men, the talent and experience requisite being transmitted from sire to son for generations. The great hotels now in their competent hands are no less than seven in number: the Ocean House, Long Branch; the Sturtevant House, New York; the Eutaw House, Baltimore; the Delevan House, Albany; the Clarendon House, Saratoga; the Leland House, Springfield, Illionis, and the new Palace Hotel, San Francisco—the largest hotel in the world. These seven hotels are capable of comfortably housing 3,500 guests. Surely a family equal to the task of supplying a home for an army like this, must have its full share of talent and skill.

Long Branch is beautiful. Its beach is 12 miles long, and green grass grows close down to the margin of the high bluff, against which the ocean beats at high water. The drive along the bank is vastly superior to any ocean drive which we have ever seen.

Mr. Warren Leland, Jr., is making a specialty of sea-foods at the Ocean House. Two fishermen are constantly employed to wring from old ocean her choicest supplies. Added to the fish-foods, he has introduced a valuable bread, made from choice wheat meal and sea-water. It is a very valuable agent for the relief of debilitated conditions and impoverishment of the blood. Weak and sickly women and puny infants are speedily restored to vigorous health by the use of Mr. Leland's Sea-Water Biscuit.

Our investigations into the subject of summer resorts for the pent-up residents of great cities did not terminateatLongBranch, but space is wanting for further notes. We may not have suggested to any reader, the answer to the question with which we commenced, but we have thrown out some hints in these two articles, which we doubt not will be heeded by many. Some of the places which we have named, we hope to see again before the summer is ended; and we may hereafter convey to our readers the conclusions which reach us on more intimate acquaintance with some of the attractive spots herein alluded to.

